# Biomimetic Strategies of Slip Sensing, Perception, and Protection in Prosthetic Hand Grasp

**DOI:** 10.3390/biomimetics9120751

**Published:** 2024-12-11

**Authors:** Anran Xie, Zhuozhi Zhang, Jie Zhang, Tie Li, Weidong Chen, James Patton, Ning Lan

**Affiliations:** 1 School of Biomedical Engineering, Shanghai Jiao Tong University, Shanghai 200030, China; anranx@sjtu.edu.cn (A.X.);; 2 i-Lab Suzhou Institute of Nano-Tech and Nano-Bionics, Chinese Academy of Sciences, Suzhou 215123, China; tli2014@sinano.ac.cn; 3 Department of Automation, Institute of Medical Robotics, Shanghai Jiao Tong University, Shanghai 200240, China; wdchen@sjtu.edu.cn; 4 The Richard and Loan Hill Department of Biomedical Engineering, University of Illinois Chicago, Chicago, IL 60607, USA; pattonj@uic.edu

**Keywords:** slip sensor, bi-state sensory feedback, biomimetic sensorimotor control, prosthetic hand, slip prevention

## Abstract

This study develops biomimetic strategies for slip prevention in prosthetic hand grasps. The biomimetic system is driven by a novel slip sensor, followed by slip perception and preventive control. Here, we show that biologically inspired sensorimotor pathways can be restored between the prosthetic hand and users. A Ruffini endings-like slip sensor is used to detect shear forces and identify slip events directly. The slip information and grip force are encoded into a bi-state sensory coding that evokes vibration and buzz tactile sensations in subjects with transcutaneous electrical nerve stimulation (TENS). Subjects perceive slip events under various conditions based on the vibration sensation and voluntarily adjust grip force to prevent further slipping. Additionally, short-latency compensation for grip force is also implemented using a neuromorphic reflex pathway. The reflex loop includes a sensory neuron and interneurons to adjust the activations of antagonistic muscles reciprocally. The slip prevention system is tested in five able-bodied subjects and two transradial amputees with and without reflex compensation. A psychophysical test for perception reveals that the slip can be detected effectively, with a success accuracy of 96.57%. A slip protection test indicates that reflex compensation yields faster grasp adjustments than voluntary action, with a median response time of 0.30 (0.08) s, a rise time of 0.26 (0.03) s, an execution time of 0.56 (0.07) s, and a slip distance of 0.39 (0.10) cm. Prosthetic grip force is highly correlated to that of an intact hand, with a correlation coefficient of 96.85% (2.73%). These results demonstrate that it is feasible to reconstruct slip biomimetic sensorimotor pathways that provide grasp stability for prosthetic users.

## 1. Introduction

The human hand contains rich tactile receptors and responsive sensorimotor control loops that relay information about grip force, friction force, shape, and texture to the central nervous system (CNS) through the spinal cord [1,2,3,4]. The CNS then generates descending alpha motor commands to facilitate proper grasping [5,6]. When the grip force is insufficient, slip activates mechanoreceptors in the fingertips and triggers a stereotyped hierarchical sensorimotor sequence involving short-latency spinal reflex, long-latency spinal reflex, and a voluntary reaction to ensure the dynamic coupling of grip and load forces to prevent slip [7,8,9,10]. Short-latency spinal reflex acts out automatic and subconscious motor actions in the fastest way. Long-latency spinal reflex incorporates feedback from both the spinal cord and higher brain regions as an intermediate step. Delayed voluntary response is initiated by higher-level brain regions, allowing for more deliberate and precise control of grip force. However, individuals with an amputation lose most of these sensorimotor pathways, impairing their ability to perceive and protect their grasp promptly. Reconstructing the slip sensorimotor pathways is critical for ensuring grasp safety in daily manipulation for prosthetic users.

Many studies aim to reconstruct slip sensorimotor pathways for amputees. Slip signal sensing typically utilizes multi-axis force sensors to measure normal and shear forces, applying Coulomb’s law or friction cone models to establish mechanical relationships [11,12]. Alternatively, slip is detected as a high-frequency signal using time–frequency transformations, filtering, and differentiation [13,14,15,16,17]. These methods detect slip events effectively but rely on specific friction coefficients or slip thresholds. When contacted objects change, corresponding adjustments are needed and pose practical challenges in real-world scenarios. For slip perception, several studies transmit slip information to amputees through neural stimulation electrodes or electro-vibration feedback [12,18,19,20,21]. Subjects exhibit high slip identification rates and adjust grip force voluntarily to prevent object slip. However, current stimulation methods mainly trigger voluntary adjustments, thus neglecting potential reflex grasp adjustments. For slip control, approaches such as PID, fuzzy, and sliding mode control adjust the desired force or position in real time [17,22,23,24] but struggle with accurate target grip force prediction. Most current slip research still lacks alignment with human physiology [8], so understanding the human slip sensorimotor mechanisms could provide insights for reconstructing sensorimotor pathways in amputees.

The human hand seamlessly integrates tactile information from shear and normal forces and combines reflexive and voluntary control, which provides a template for prosthetic solutions. As prosthetic hand research has advanced, more techniques have been developed to mimic the human hand’s sensorimotor pathways. In non-invasive sensory feedback, transcutaneous electrical nerve stimulation (TENS) applied to the projected finger map areas in amputees can evoke natural finger sensations, which are called evoked tactile sensations. These somatotopically evoke tactile sensations, enabling amputees to regain the ability to recognize the physical properties and grip force of objects [25,26,27]. This sensory feedback strategy provides a promising natural approach for restoring slip sensory pathways in amputees. In terms of prosthetic hand control, recent studies have introduced a neuromorphic control strategy [28,29,30], utilizing antagonistic muscle models with human-like biomechanical compliant characteristics to significantly enhance control capabilities. This approach lays the foundation for replicating short-latency reflex and voluntary compliance control in prosthetic hands.

This study aims to mimic the human hand’s neurophysiological mechanisms for slip sensing, perception, and protection and restore the slip sensorimotor pathways between prosthetic hands and users. First, we introduce a biomimetic slip detection method using a novel slip sensor. Second, we design a TENS bi-state sensory feedback strategy for force and slip perception and enable voluntary control. Third, we leverage a biomimetic short-latency reflex loop that is integrated with the voluntary control of subjects to achieve slip prevention. Finally, tests of psychophysical perception and slip protection are conducted on two amputees and five able-bodied subjects to validate the feasibility of the biomimetic sensorimotor strategies for slip sensing, perception, and protection.

## 2. Materials and Methods

### 2.1. Biomimetic Sensorimotor Prosthetic Hand System

The prosthetic hand used in this study was based on an open-source design, InMoov [31]. Each finger had four phalanges with two 0.5 mm radius tendons to mimic antagonistic muscles with single-degree-of-freedom movement, controlled by two linear-force servo-motors with proportional plus integral (PI) control using the Ziegler–Nichols method [32]. The maximum fingertip force was 20 N. The motor provided a 3 cm drive distance and a maximum force of 70 N (LAF 30, Inspire Robotics Inc., Beijing, China). This system had a cutoff frequency of 7.96 Hz and a phase shift range of 2 to 205 degrees [29]. Antagonistic muscle models were implemented using Very Large Scale Integration (VLSI) chips (Spartan-6, Xilinx, San Jose, CA, USA) capable of real-time calculations at 1 kHz. Target muscle forces were transmitted via Bluetooth to a PI control board for finger control.

**Figure 1:**
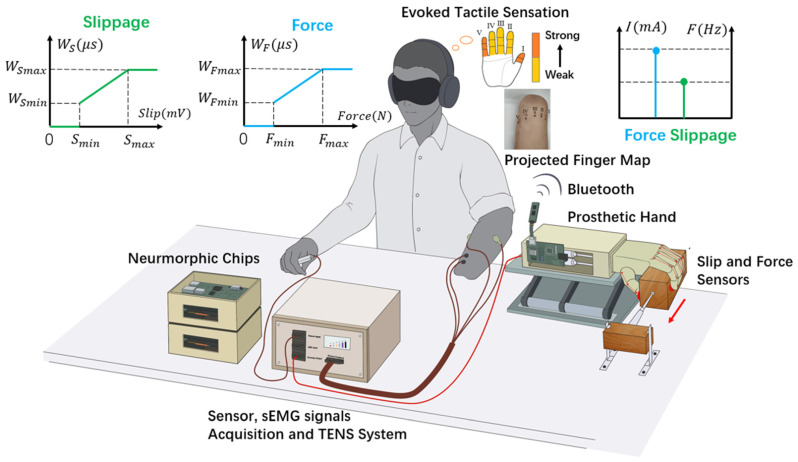
Biomimetic sensorimotor prosthetic hand system. An amputee using sEMG signals to control a prosthetic hand to grasp a wooden block, which was pulled by a motor to simulate slip. The antagonistic muscle model simulated muscle-compliant biomechanics and subconscious short-latency spinal reflex. A neuromorphic chip calculated muscle forces in real time and transmitted them via Bluetooth to the prosthetic hand. Fingertip sensors collected force and slip information. A TENS system encoded force and slip sensor information into two electrical stimulation signals applied to the projected finger map to evoke buzz and vibration tactile sensations. These bi-state sensory feedback and compliant control strategies enable the amputee to perceive and protect against slip during grasping.

The system also included surface electromyography (sEMG) and sensor acquisition components, as well as a programmable TENS unit. sEMG signals were filtered and output at 1 kHz. Two force sensors (2.5 cm * 1.4 cm, response time < 5.0 μs, Flexiforce, Tekscan Inc., Boston, MA, USA) on the index and thumb fingers, along with a slip sensor on the thumb finger, sampled data at 100 Hz. The TENS system encoded these signals into biphasic, charge-balanced pulse sequences via custom Ag/AgCl electrodes to the projected finger map areas, as shown in Figure 1 [33]. This TENS evoked sensations through direct neural pathways to the primary sensory cortex, allowing amputees to perceive slip and grip events somatotopically [25].

### 2.2. Slip Sensor Fabrication and Signal Processing

**Figure 2:**
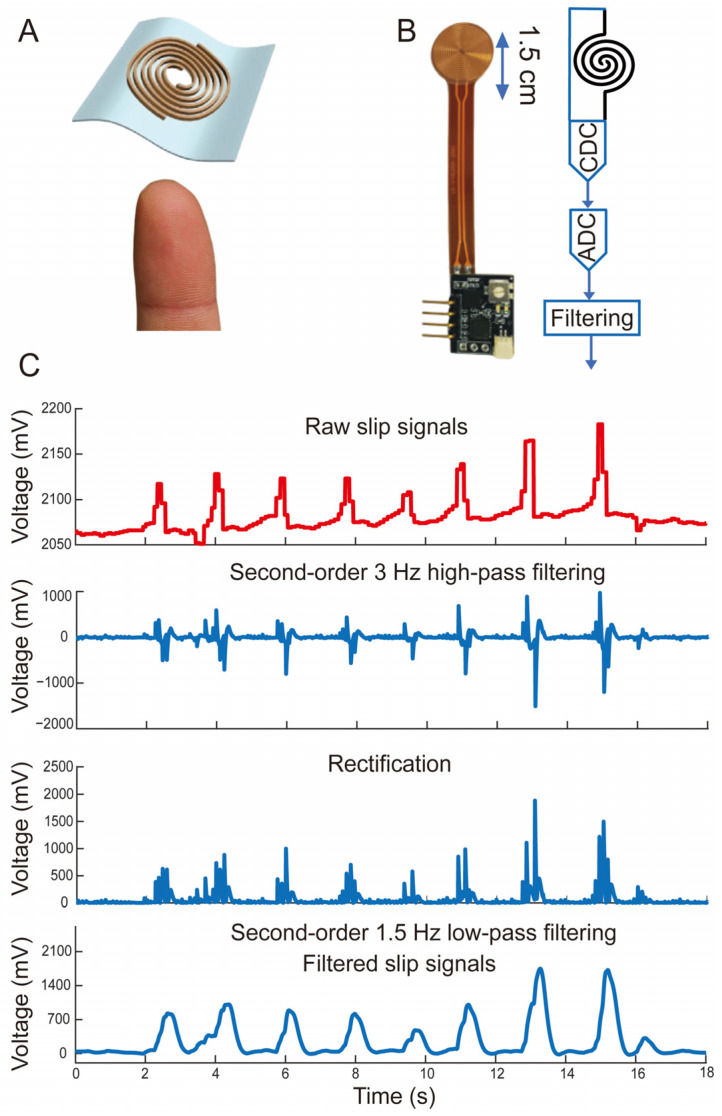
Slip sensor structure and signal processing. (**A**) Slip sensor structure diagram and human fingerprint. (**B**) Slip sensor with integrated circuit and signal processing diagram. CDC and ADC are short for capacitance-to-digital converter and analog-to-digital converter, respectively. (**C**) Slip signal filtering process.

This novel slip sensor, with a 1.5 cm diameter, mimicked Ruffini endings [34]. It was inspired by human fingerprints and constructed as a spiral–planar parallel capacitor with an intermediate dielectric layer and two capacitor plates (Figure 2A). The plates were made of silver nanowire 3D polydimethylsiloxane (PDMS) electrodes and had a fixed width of 15 µm and a spacing of 50 µm. This symmetrical design ensured consistent sensitivity to shear forces in any direction on the plane. Based on this novel structure, the sensor capacitance remained nearly constant under various applied normal forces but decreased with sliding. Previous tests demonstrated that the capacitance remained stable under 0–20 N normal forces but changed exponentially from 0 to 40 pF as the sliding friction force increased from 0 to 10 N. This selective response mimicked the physiological properties of Ruffini endings and led to slip events being effectively detected. Details on sensor fabrication and testing are available in the previous literature [34].

To enhance signal stability, a capacitor voltage converter and filtering were used to minimize environmental noise. The specific filtering steps were as follows. Firstly, second-order 3 Hz high-pass filtering was performed to remove low-frequency components and retain sudden-slip high-frequency components. Then, rectification was carried out to facilitate subsequent processing and analysis. Finally, second-order 1.5 Hz low-pass filtering was implemented to remove high-frequency noise and further smooth the signal; in this way, the slip signals were reliably detected, as shown in Figure 2C.

### 2.3. Slip-Force Bi-State Sensory Feedback

This study utilized TENS based on projected finger map areas to restore the ascending sensory pathways for slip and force perception for amputees. Buzz sensation was chosen to encode force signals due to its wide pulse width modulation range and high sensitivity [33]. A vibration sensation was used to encode slip signals, as prior research has shown its effectiveness in conveying slip information [21,35,36,37]. We designed a bi-state sensory coding strategy by adjusting the pulse width, amplitude, and frequency of the TENS system. When no slip event occurred, buzz stimulation was applied to the index and thumb fingers, allowing subjects to perceive grip force. Upon slip detection, the stimulation immediately switched to the vibration modality, enabling slip perception through the change in sensations.

Prior studies identified 50 Hz for buzz and 20 Hz for vibration modality as dominant frequencies [26,33]. We fixed these frequencies and evaluated the corresponding amplitude and pulse width. The amplitude started at 1 mA and increased in 0.5 mA steps until the buzz or vibration sensation was clearly perceived. The pulse width began at 20 µs and increased in 20 µs increments to determine the perception threshold. The minimum pulse width was defined as the threshold for initial perception ($W_{Fmin},W_{Smin}$), while the maximum pulse width was set when the sensation changed ($W_{Fmax},W_{Smax}$). Each stimulation lasted 3 s, followed by a 5 s rest. The maximum and minimum grip forces ($F_{max},F_{min}$) and slip sensor outputs ($S_{min},S_{max}$) were linearly mapped to the pulse width ranges for both force and slip signals using Equations (1) and (2).

(1) $$\left\{\begin{array}{c} W_{F}=0,\;F<F_{min}\\ W_{F}=\frac{W_{Fmax}-W_{Fmin}}{F_{max}-F_{min}}*\left(F-F_{min}\right)+W_{Fmin},\;F_{min}\leq F<F_{max}\\ W_{F}=W_{Fmax} \end{array}\right.$$

(2) $$\left\{\begin{array}{c} W_{S}=0,\;S<S_{min}\\ W_{S}=\frac{W_{Smax}-W_{Smin}}{S_{max}-S_{min}}*\left(S-S_{min}\right)+W_{Smin},\;S_{min}\leq S<S_{max}\\ W_{S}=W_{Smax} \end{array}\right.$$

### 2.4. Voluntary and Reinforcement Controls

**Figure 3:**
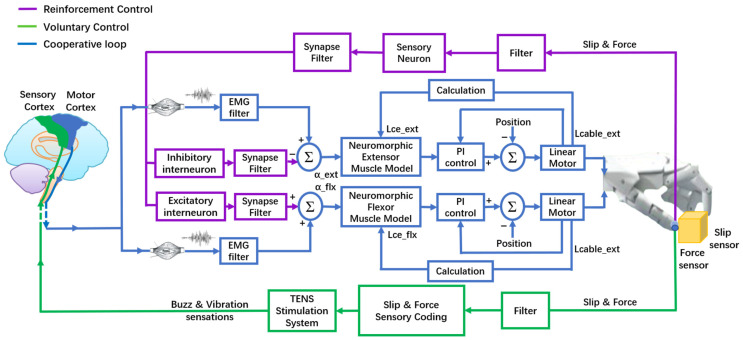
Schematic diagram of biomimetic sensorimotor prosthetic hand for slip sensing, perception, and protection through voluntary and reinforcement controls. A slip sensor that responded only to shear force transmitted the slip signals to the amputees via a TENS system. Using a bi-state sensory feedback strategy, amputees perceived both slip events and grip force. The antagonistic muscle models and neuromorphic reflex loop calculated the muscle forces in real-time, allowing amputees to adjust grip force to prevent slip in voluntary or reinforcement controls.

To simulate the stereotypical sequence of short-latency reflex and voluntary control [9], we designed biomechanical descending control pathways that integrated both voluntary and reinforcement controls (Figure 3). Voluntary control was defined as the process in which subjects voluntarily generate grip force based on the force sensory feedback, as represented in the green and blue lines in Figure 3. sEMG signals were normalized during rest and maximum voluntary contraction to generate alpha motor commands for activating 768 motor neurons using the Henneman size principle [38]. Neuron spikes were then activated using antagonistic Hill-type muscle models to calculate muscle forces using the sEMG activation level (α) and muscle fascicle length ($L_{ce}$) [39,40]. Compliant voluntary control was achieved through these muscle force–length properties. Previous studies have achieved coupling between motor cable length ($L_{cable}$) and muscle fascicle length and validated the muscle characteristics [28,29,30].

**Figure 4:**
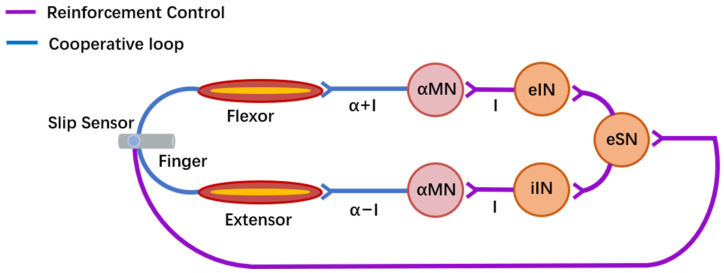
Neuromorphic reflex loop in reinforcement control. A sensory neuron and interneurons were added to simulate the short-latency reflex, which automatically increased grip force along with the slip signals. αMN, eIN, iIN, eSN, and I represent the alpha motor neuron, excitatory and inhibitory interneurons, excitatory sensory neuron, and synapse current, respectively.

Reinforcement control is represented as purple and blue lines in Figure 3. It was defined as a combination of voluntary control with a neuromorphic short-latency reflex strategy. We incorporated the Izhikevich neuron model with a neuromorphic sensory neuron and interneurons to reinforce voluntary control, as shown in Figure 4 [41,42]. Filtered slip signals (I) were mapped linearly to input currents (0.0–35.0 mA) for a fast-spiking excitatory sensory neuron, which generated membrane potentials transmitted via a synapse model. The parameters in (3) for the sensory neuron model were *a* = 0.1 ms^−1^, *b* = 0.2 ms^−1^, *c* = −65 mV, and *d* = 2 mV/ms. Then, the synapse generated the sensory neuron postsynaptic current (I), which flowed to low-threshold excitatory and inhibitory interneurons, which were also used in the Izhikevich neuron model with different parameters; the parameters in (3) for the interneuron model were *a* = 0.02 ms^−1^, *b* = 0.25 ms^−1^, *c* = −65 mV, and *d* = 2 mV/ms.

(3) $$\left\{\begin{array}{c} \frac{dV}{dt}=0.04V^{2}+5V+140-U+I\\ \frac{dU}{dt}=a(bV-U) \end{array}\right.\;\mathrm{if}\;V\geq 30\;\mathrm{mV},\;\mathrm{then}\;\left\{\begin{array}{c} V\leftarrow c\\ U\leftarrow U+d \end{array}\right.$$

The synapse model was proposed by Glowatzki and was used in this study [43]. This model depicted the process of a single neuron being fired in exponential form, showing the postsynaptic current (*I*) dynamic behavior of the neuron’s activity over the activation time (*t*). Modified synapse rise and fall time parameters ($\tau_{r}$ = 0.08 s, $\tau_{f}$ = 0.19 s, $V_{m}$ = 5.2 mV) allowed the interneurons to produce postsynaptic currents, as shown in (4). These synaptic computations were realized through filtering by a host computer and normalized as reflex compensation, as shown in Figure 4. Excitatory postsynaptic currents increased the flexor motor neuron output, while inhibitory interneurons reduced the extensor motor output. The subjects adjusted the sEMG signals for voluntary control guided by TENS sensory feedback, while reinforcement control simultaneously enhanced grip force in response to slip signals.

(4) $$I\left(t\right)=\left\{\begin{array}{c} V_{m}\left(e^{-\frac{t}{\tau_{f}V_{m}}}-e^{-\frac{t}{{\tau_{r}V}_{m}}}\right),\;\mathrm{if}\;t\geq 0\\ 0,\;\mathrm{otherwise} \end{array}\right.$$

### 2.5. Experimental Protocols

To validate the slip sensor detection accuracy and assess the subjects’ ability to perceive and protect against slip based on current sensorimotor strategies, psychophysical perception and slip protection tests were designed. Two subjects with a left unilateral transradial amputation (A1–A2, male, aged 64 ± 2 years, both lacking prior experience using myoelectric prosthetic hands) and five able-bodied subjects (S1–S5, three females, two males, aged 27 ± 2 years) participated in all the experiments. The two amputees had evoked tactile sensations and were willing to participate in this study. This study was approved by the Institutional Review Board for Human Research Protections, Shanghai Jiao Tong University (IRB number: E2020021I). All subjects were informed about the experimental procedure and signed an informed consent form prior to the experiment.

#### 2.5.1. Psychophysical Perception Test

We first evaluated the detection accuracy of the slip sensor. The prosthetic hand without an applied grip force came into contact with a wooden block (5 * 5 * 10 cm^3^) placed on a table (Figure 1). The block was connected to a PI force-controlled motor that applied horizontal constant load forces in 350 randomized trials. In 175 trials, the motor applied a force of 5 N, ensuring 100% slip occurrence to measure the true positive and false positive detection rates. The remaining 175 trials used a 0 N force to test the true negative and false negative detection rates.

We then assessed the subjects’ slip perception using bi-state sensory feedback. The prosthetic hand grasped the wooden block with three fixed alpha control commands (ext = 0.1, flx = 0.3, 0.5, 0.8). The motor applied constant load forces at 15 levels (1–15 N, with 1 N step size). Subjects sat comfortably at a table with their vision and hearing blocked and detected the change in sensory feedback (Figure 1). They were instructed to press a trigger button when the perceived sensation switched from the buzz to vibration modality. No action was required if the buzz sensation remained unchanged. Each control command included 15 load force levels presented in a random order and repeated 10 times, totaling 450 trials per subject (3 control commands * 15 load force levels * 10 repetitions). The probability of slip detection was recorded and fitted using a Sigmoid function. The 50% probability point of the fitted curve, known as the point of subjective equality (PSE) [44,45], indicated the load force at which the slip detection probability reached 50%.

#### 2.5.2. Slip Protection Test

We validated the effectiveness of the slip control approach using a slip protection test under three conditions: voluntary control (VC), reinforcement control (RC), and intact hand control (IC). VC and RC aimed to replicate human sensorimotor strategies, while the IC condition served as a baseline for comparing the performance. Subjects sat comfortably at a table with visual and auditory cues blocked (Figure 1). To ensure consistent slip events, the prosthetic hand initially applied no grip force on a wooden block pulled by a PI-controlled motor at 5 N. Upon slip perception, subjects were instructed to press a trigger button and increase their grip force to hold stably within 5 s. In the IC condition, subjects wore finger covers with force sensors to ensure the prosthetic and manual control conditions had constant friction conditions. Calibration tests showed that the block stopped slipping after 3 cm of motor load force without any grip force. Grip force was the sum of forces on the index and thumb fingers. Each condition was randomized and repeated 10 times, totaling 30 trials per subject.

### 2.6. Data Processing and Statistical Analysis

To evaluate the control performance of these slip sensorimotor strategies, we measured the following six indexes:

(1) Short-latency reflex time:

The time from slip detection to the interneurons generating reflex neuron compensations.

(2) Voluntary response time:

The time from slip detection to when the subject pressed the trigger button.

(3) Grip force rise time:

The time required for grip force to increase from 10% to 90% of the target force, where the target force was the average force during the last 3 s of the hold phase.

(4) Grip force execution time:

The time from slip detection to when grip force reached 90% of the target force.

(5) Grip force correlation:

The Pearson correlation coefficient was calculated to compare the grasp performance of a prosthetic hand to that of an intact hand (5). PH represented the VC or RC condition with the prosthetic hand, and IH represented the IC condition. $\bar{PH}$ and $\bar{IH}$ denoted the average grip force under prosthetic and intact hand conditions, respectively. ${PH}_{i}$ and ${IH}_{i}$ referred to individual force data points in each trial. A correlation was generated for each trial T under VC or RC conditions.

(5) $$\gamma =\frac{\sum_{t=0}^{t=T}\left({PH}_{i}-\bar{PH})({IH}_{i}-\bar{IH}\right)}{\sqrt{\sum_{t=0}^{t=T}{\left({PH}_{i}-\bar{PH}\right)}^{2}\sum_{t=0}^{t=T}{\left({IH}_{i}-\bar{IH}\right)}^{2}}}$$

Each subject’s maximum voluntary grip force was tested using both their intact and prosthetic hands to set normalization standards for all control conditions. Since the data did not pass the Kolmogorov–Smirnov test, the non-parametric Kruskal–Wallis test was used for statistical analysis, followed by Bonferroni correction for post hoc comparisons to assess group differences. All statistical analyses were conducted using IBM SPSS Statistics 26.0. Control programs ran on Visual Studio 2022, and data analysis was conducted in MATLAB R2020b. Data distributions were presented as box plots showing the median and interquartile range (IQR) in parentheses. Red points represent outliers, and black lines indicate the minimum, maximum, quartiles, and median values. Individual subject performance was represented by the median value in the overall group analysis.

## 3. Results

### 3.1. Validation of Neuromorphic Short-Latency Reflex

**Figure 5:**
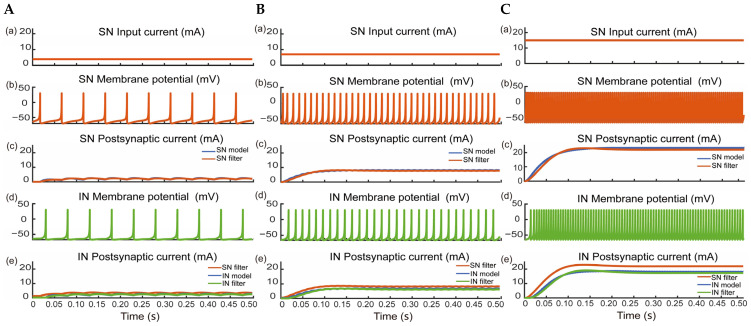
Reflex response in neuromorphic short-latency reflex. (**A**) Reflex response with a minimum input current of 3.9 mA from the sensory neuron (SN) to the interneuron (IN). The input current, membrane potential, and postsynaptic current generated by the synapse model and filter of the SN and IN are displayed sequentially (**a**–**e**). (**B**) Reflex response with input currents of 7.0 mA. The meaning of (**a**–**e**) are same with (**A**). (**C**) Reflex response with input currents of 15.0 mA. The meaning of (**a**–**e**) are same with (**A**).

We simulated slip signals through the neuromorphic reflex loop using three constant input currents and compared the response of the synapse filter and synapse model. Figure 5A(a) shows a 3.9 mA minimum input current triggering the reflex response, generating membrane potential in the sensory neuron (Figure 5A(b)). The synapse model and filter then produced sensory neuron postsynaptic currents (Figure 5A(c)). These currents activated the interneuron, generating membrane potential (Figure 5A(d)) and further interneuron postsynaptic currents were produced by the synapse model and filter (Figure 5A(e)). Figure 5B,C display the responses for 7.0 mA and 15.0 mA input currents, where both the membrane potential and postsynaptic currents increased proportionally, with the synapse filter closely matching the synapse model. Short-latency reflex times of 30.0 ms, 17.5 ms, and 14.5 ms also shortened as the reflex activation increased for input currents of 3.9, 7.0, and 15.0 mA, respectively, all close to the range of neurophysiological short-latency spinal reflexes (~25–50 ms) [8]. These results confirmed the feasibility of this neuromorphic short-latency reflex.

### 3.2. Results of Psychophysical Perception Test

**Figure 6:**
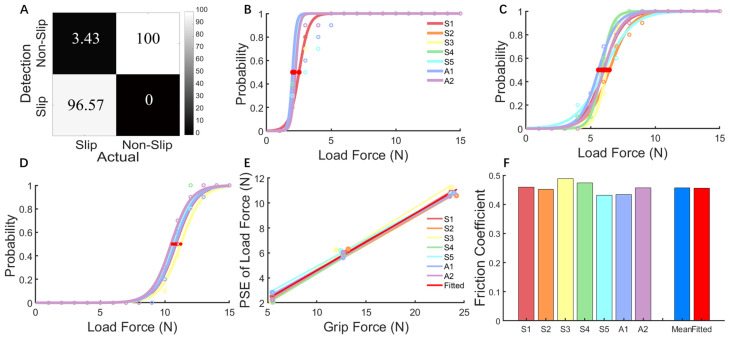
Results of the psychophysical perception test. (**A**) Slip sensor’s true positive, true negative, false positive, and false negative sensing rates. (**B**–**D**) Probability of perceiving slip events and fitted psychophysical curves for seven subjects at three grip force levels (ext = 0.1, flx = 0.3, 0.5, 0.8); red dots indicate points of subjective equality (PSE) of load force, where the probability of perceiving a slip event was 50%. (**E**) PSE for perceiving slip events at three grip force levels for all subjects. (**F**) Friction coefficient fitted from the PSE of load forces and three grip force levels for individual subjects and the subjects overall.

The confusion matrix in Figure 6A shows this slip sensor with a true positive sensing rate of 96.57%, a true negative rate of 100.00%, a false positive rate of 0.00%, and a false negative rate of 3.43%. The psychophysical perception test results demonstrated high consistency and stability in slip perception across various grip and load forces, as reflected in the fitted PSE values and friction coefficients. Figure 6B–D reveal that under three grip force levels, the probability of sensing slip events increased with greater load forces. At a lower grip force (flx = 0.3, Figure 6B), the seven subjects had lower PSE values of load force, indicating that slip events occurred and were detected at smaller load forces. At a higher grip force (flx = 0.8, Figure 6D), the increased PSE values signified that greater load forces were needed for slip occurrence. Figure 6E presents the PSE values of load force across three grip force levels, with the slope of these fitted relationships representing the individual friction coefficient for each subject. The overall friction coefficient obtained from these 21 fitted PSE values of load force (7 subjects * 3 grip force levels) across all subjects was 0.46 (Figure 6F), consistent with the average coefficient from the seven subjects and aligning with values in the literature [5,11,46]. These results confirmed the feasibility of reconstructing the slip perception pathways using the TENS bi-state sensory feedback strategy.

### 3.3. Results of Slip Protection Test

**Figure 7:**
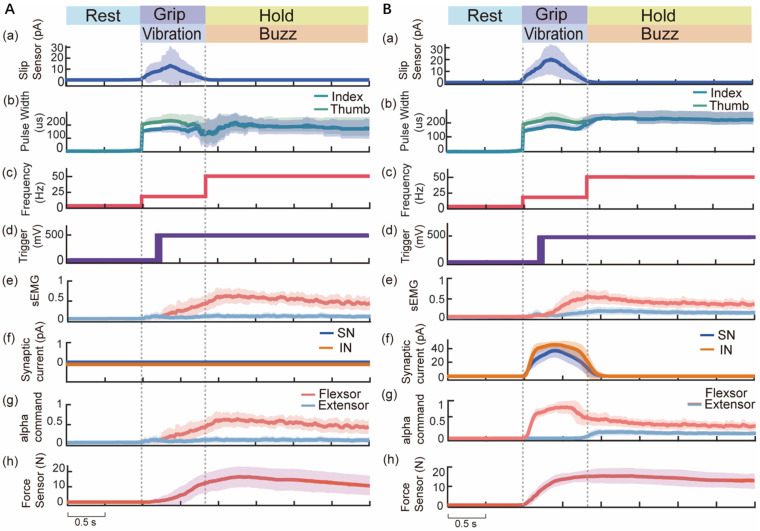
Processes of voluntary and reinforcement control in an amputee (A1) during the slip protection test. (**A**) Voluntary control. The amputee perceived slip events through bi-state sensory feedback and subsequently increased the sEMG signals and grip force. Changes in signals from the slip sensor, stimulation pulse width and frequency, trigger button, sEMG signals, synaptic current, normalized alpha control command, and grip force are displayed sequentially (**a**–**h**). Dark lines represent the average values across 10 trials, while the lightly shaded areas indicate the average value ± standard deviation. In the reinforcement control condition (**B**), after slip events were detected (**a**), the stimulation pulse with and frequency were changed (**b**–**c**), A1 pressed the trigger botton (**d**). The sensory neuron (SN) and interneuron (IN) generated postsynaptic currents (**f**) that influenced the antagonistic alpha control command (**g**). Simultaneously, the amputee gradually increased the sEMG signals through electrical stimulation, leading to a rapid increase in grip force (**e**–**h**).

Figure 7 illustrates the processes of voluntary and reinforcement control in a subject with an amputation (A1) during the slip protection test. In the rest phase, there was no response from the force and slip sensors, nor any stimulation. Upon detecting a slip event, the TENS system delivered electrical stimulation in the vibration modality to the amputee’s projected finger areas, with pulse width linearly encoding the slip signals (Figure 7A(a–c)). The amputee then pressed a button to record the response time (Figure 7A(d)) and increase the sEMG signals and grip force to prevent the block from slipping (Figure 7A(e–h)). During the stable hold phase, the stimulation switched to the buzz modality, with pulse width linearly encoding the grip force. Figure 7B shows this amputee’s performance under reinforcement control, perceiving slip events through the vibration sensation (Figure 7B(a–c)) and pressing the button (Figure 7B(d)). The neuromorphic short-latency reflex compensated for perception processing, generating postsynaptic currents in a sensory neuron and interneurons, leading to increased flexor commands and decreased extensor commands (Figure 7B(f,g)). The amputee then voluntarily increased the sEMG signals after perceiving this slip event. Consequently, the grip force increased rapidly with reflex compensations and voluntary adjustments (Figure 7B(e–h)).

**Figure 8:**
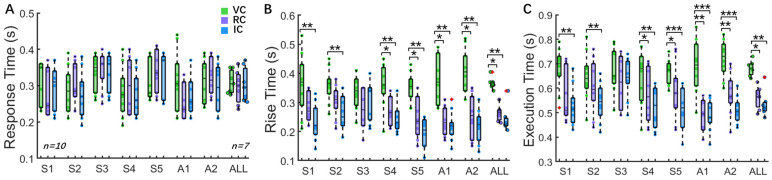
Time indexes for individual subjects and the overall group under three control conditions. (**A**) Response time. Each individual subject had 10 data points (n = 10 trials), and the overall results represent the median of the 7 subjects (n = 7 subjects). (**B**) Rise time. (**C**) Execution time. Significance is indicated as * (*p* < 0.05), ** (*p* < 0.01), and *** (*p* < 0.001). VC, RC, and IC represent voluntary control, reinforcement control, and intact hand control, respectively.

We analyzed the time indexes under VC, RC, and IC conditions. As shown in Figure 8A, there was no significant difference in response time across the three conditions, with a median response time of 0.30 s for all subjects (VC: 0.31 s, 0.06 s; RC: 0.30 s, 0.08 s; IC: 0.30 s, 0.09 s). Subjects reliably perceived slip events using bi-state sensory feedback with no significant difference from the IC condition, confirming the effectiveness of reconstructing slip perception pathways for amputees. In the VC condition, the need to voluntarily increase grip force after perceiving slip events resulted in a slower rise time (VC: 0.37 s, 0.01 s) compared to the RC (0.26 s, 0.03 s) and IC (0.22 s, 0.03 s) conditions (Figure 8B), as demonstrated clearly in Figure 7A(h) and Figure 7B(h). In the RC condition, the neuromorphic short-latency reflex facilitated delayed perception processing, achieving a rise time comparable to that observed in the IC condition. The execution time followed a similar trend, with the RC condition (0.56 s, 0.07 s) showing no significant difference from the IC condition (0.52 s, 0.03 s) but being faster than VC condition (0.68 s, 0.04 s). These results confirmed the feasibility of reconstructing the slip control pathways using reinforcement and voluntary responses.

**Figure 9:**
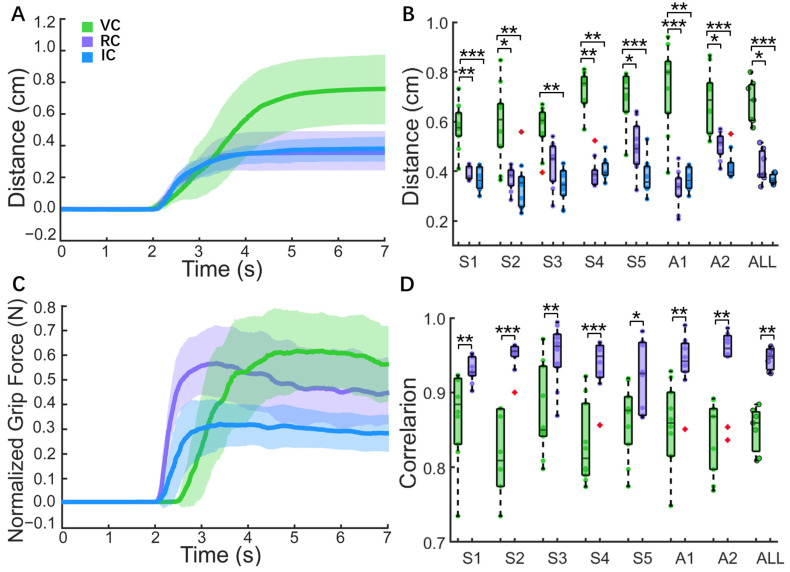
Analysis of slip distance and grip force correlation. (**A**) Representative slip distance for an amputee (A1) under three control conditions. (**B**) Slip distance for individual subjects and overall group under the three control conditions. Each subject had 10 data points (n = 10 trials), and the overall results represent the median value of the 7 subjects (n = 7 subjects). (**C**) Representative normalized grip force for A1 under the three control conditions. (**D**) Grip force correlation for individual subjects and the overall group under the voluntary and reinforcement control conditions. Significance is indicated as * (*p* < 0.05), ** (*p* < 0.01), and *** (*p* < 0.001).

The slip distance and grip force correlation further confirmed these reconstructed sensorimotor feasibilities. Figure 9A shows the representative slip distance from the amputee (A1) under three control conditions. The IC condition resulted in the shortest slip distance. Across all subjects, the median slip distance was minimal in IC conditions (0.36 cm, 0.03 cm) and similar under RC conditions (0.39 cm, 0.10 cm), with no significant difference (Figure 9B). The VC condition showed a longer slip distance (0.69 cm, 0.14 cm), which was significantly different from that of the RC and IC conditions. Figure 9C shows that the representative normalized grip force increased faster in the IC and RC conditions, while the VC condition exhibited a delay. The grip force correlation under the RC condition (96.85%, 2.73%) was significantly higher than under the VC condition (85.91%, 5.23%), as shown in Figure 9D, demonstrating the effectiveness of reflexive reinforcement control and its alignment with natural hand slip control performance. Detailed performance indexes for this slip protection test for the subjects overall are summarized in Table 1.

## 4. Discussion

Many studies have proposed prosthetic slip protection solutions [13,17,22,35,36,37], but they still remain far from replicating the neurophysiological mechanisms and effectiveness of the human hand [1,2,3,4,5,6]. This study developed a biomimetic sensorimotor prosthetic hand driven by a novel slip sensor. The system detected slip events without complex calculation and used TENS bi-state sensory feedback combined with a slip reflex strategy to allow subjects to prevent slip in either voluntary or reinforcement control. The results showed that the slip sensor achieved high detection accuracy. The subjects clearly perceived slip events under varying grip and load force conditions and voluntarily increased the grip force and reduced the slip distance. In the reinforcement control approach, the response speed and slip distance were comparable to those of an intact hand, confirming that this prosthetic hand successfully replicated the neurophysiological process and effectiveness from the short-latency reflex to the voluntary response.

This novel slip sensor effectively sensed slip events for amputees, overcoming the limitations of traditional methods, which often rely on setting friction coefficients or complex signal calculations [13,17]. This slip sensor was only sensitive to shear forces, filtering out irrelevant external forces. Upon the onset of slip, it swiftly captured changes in shear forces and outputted the slip spike pulses through filtering and amplification (Figure 2C). These signal pulses conveyed temporal and intensity characteristics for subsequent slip perception and reflex control. The results showed that the sensor’s detection accuracy reached 96.57%, with a false negative rate of 3.43% and no false positive rate (Figure 6A). This slip detection approach improved real-time detection and reliability, offering a practical solution for future slip sensor research and applications.

The TENS bi-state sensory feedback strategy successfully restored the amputees’ perceptions of slip and force. The evoked tactile sensations of buzz and vibration were highly consistent with natural finger perception, showing no significant difference in slip response time compared to the intact hand (Figure 8A). The median response time for all subjects was 0.30 s, which was slightly slower than the neurophysiological voluntary response time (0.10 s) [8], possibly due to a lack of neural plasticity for these sensory modalities. Additionally, the finger gloves may have affected intact hand tactile perception. The psychophysical perception test demonstrated consistent differentiation between stable grasp and slip events under varying grip and load forces (Figure 6B–F). The subjects adjusted their grip force with lower cognitive load, a high response speed, and minimal slip distance to prevent further slip (Figure 7). This bi-state sensory strategy ensured consistent perception aligned with grasp events. It has great potential for dynamic grasp environments involving crushed or hot objects [27], where single-modality feedback might lead to confusion.

These biomimetic sensorimotor control strategies enabled reconstructed slip control capabilities for amputees through voluntary and reinforcement control. While TENS alone was insufficient to evoke subconscious grip reflexes, the neuromorphic short-latency reflex incorporated a sensory neuron and interneurons modeled with the Izhikevich model and synapse model. Reflex validation showed a fast-firing response with reflex times of 14.5–30.0 ms (Figure 5), aligning with the neurological reflex time range and confirming the feasibility of reinforcement control. The slip protection test showed that the median reaction and execution times with reinforcement control were similar to those of the intact hands and significantly shorter than those of the voluntary control (Figure 8B,C). Similarly, the median slip distance in the reinforcement control condition was 0.39 cm, which was notably less than the value of 0.69 cm observed under voluntary control and close to the value of 0.36 cm obtained in the intact hand condition (Figure 9A,B). The grip force correlation improved from 85.91% under voluntary control to 95.85% under reinforcement control (Figure 9C,D). These results indicate that the prosthetic hand effectively replicated the hierarchical sensorimotor control strategies. Specifically, reinforcement control enhanced grasp stability through faster reflex responses, smaller slip distances, and improved grip accuracy.

The design of voluntary control and reflex compensation strategies highlights the feasibility of replicating human-like sensorimotor control in prosthetics. Voluntary control provided more flexibility and precision, making it suitable for tasks that do not require fast action speed. For example, the subjects were able to perceive surface texture or roughness through the vibration intensity from slip signals and then adjusted the sEMG signals to fine-tune the grip force based on the object’s properties [47]. This allowed the subjects to reduce external interference and focus on the details of the task. Reinforcement control was better suited to tasks that required a secure grasp, such as handling slippery or fragile objects [20,48]. This strategy automatically adjusts grip force for swift responses to changing conditions, effectively mirroring the performance of a natural hand and providing a safer grasp experience for prosthetic users. Combining both control strategies offers a flexible and comprehensive solution for diverse tasks, enhancing the functionality and adaptability of prosthetic hands in daily use and complex grasp environments [49,50].

Despite the promising results obtained in terms of improving the slip sensing, perception, and protection of sensorimotor abilities, some limitations remained. First, only two amputees participated in this study, and more valuable results could be obtained from a larger sample size. Second, this prosthetic hand used a simple pinch grip, while a multi-finger control strategy was not addressed. Third, there were no specific guidelines for the grip force required to prevent slip, leading the subjects to tend to use the maximum grip force. It was equally important to avoid excessive grip force. Future studies will explore incorporating internal models to ensure grip force is applied within a safe margin for natural hand function [5,51,52,53], and amputees will fully wear the prosthetic hand to evaluate the effectiveness of these biomimetic sensorimotor strategies in more functional tasks.

## 5. Conclusions

This study successfully mimicked the neurophysiological mechanisms of the human hand for slip sensing, perception, and protection. This novel slip sensor enabled direct slip signal sensing, while the TENS bi-state sensory feedback strategy provided force and slip perception. The psychophysical perception results confirmed the feasibility of restoring the slip sensory pathway effectively. The slip protection tests indicated that both voluntary and reinforcement control strategies were able to prevent slip events in prosthetic hand grasping. The biomimetic reflex loop enhanced the voluntary response, leading to faster reaction times, shorter slip distances, and higher grip force correlation. This study improved the human–prosthetic interaction with slip intervention, offering a potential solution for safe and stable grasp functions in prosthetic hands.

## Figures and Tables

**Table 1:** Median and IQR for average performance of seven subjects in three control conditions.

Performance Index	Voluntary Control (VC)	Reinforcement Control (RC)	Intact Hand Control (IC)
Response time (s)	0.31, 0.06	0.30, 0.08	0.30, 0.09
Rise time (s)	0.37, 0.01 *^,††^	0.26, 0.03	0.22, 0.03
Execution time (s)	0.68, 0.04 *^,††^	0.56, 0.07	0.52, 0.03
Slip distance (cm)	0.69, 0.14 *^,†††^	0.39, 0.10	0.36, 0.03
Force correlation (%)	85.91, 5.23 **	96.85, 2.73	-

* indicated a significant difference compared to IC; ^†^ indicated a significant difference compared to RC, * at *p* < 0.05, **, ^††^ at *p* < 0.01, and ^†††^ at *p* < 0.001. - indicated that this index was not calculated.

## Data Availability

The data that support the findings of this study are available upon reasonable request from the authors.
